# Supplementary figures and images for: Host cell and viral protease targets of human SERPINs identified by in silico docking
Source: EMBO J. 2025 Sep 8;44(20):5755–84. doi: 10.1038/s44318-025-00546-6 (PMC12528359; doi:10.1038/s44318-025-00546-6)

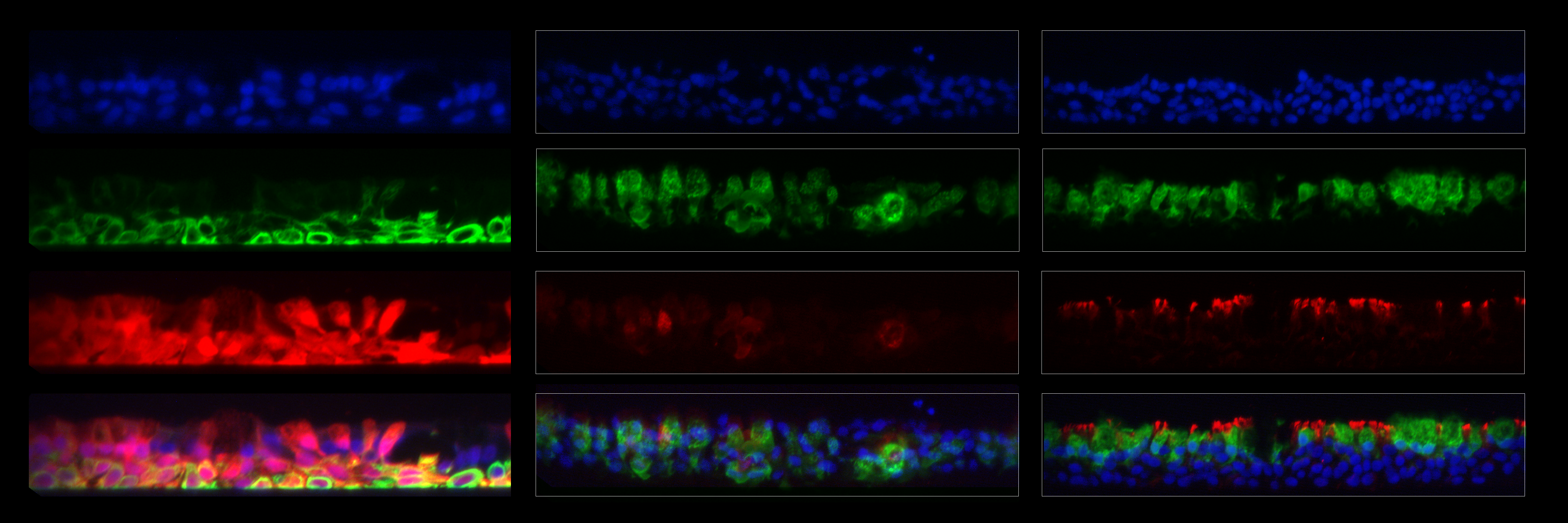

Supplement: Supplementary file 5 — Source data Fig. 2 [file 44318_2025_546_MOESM5_ESM.zip › Figure 2 resubmission of data/2A IF.tif]

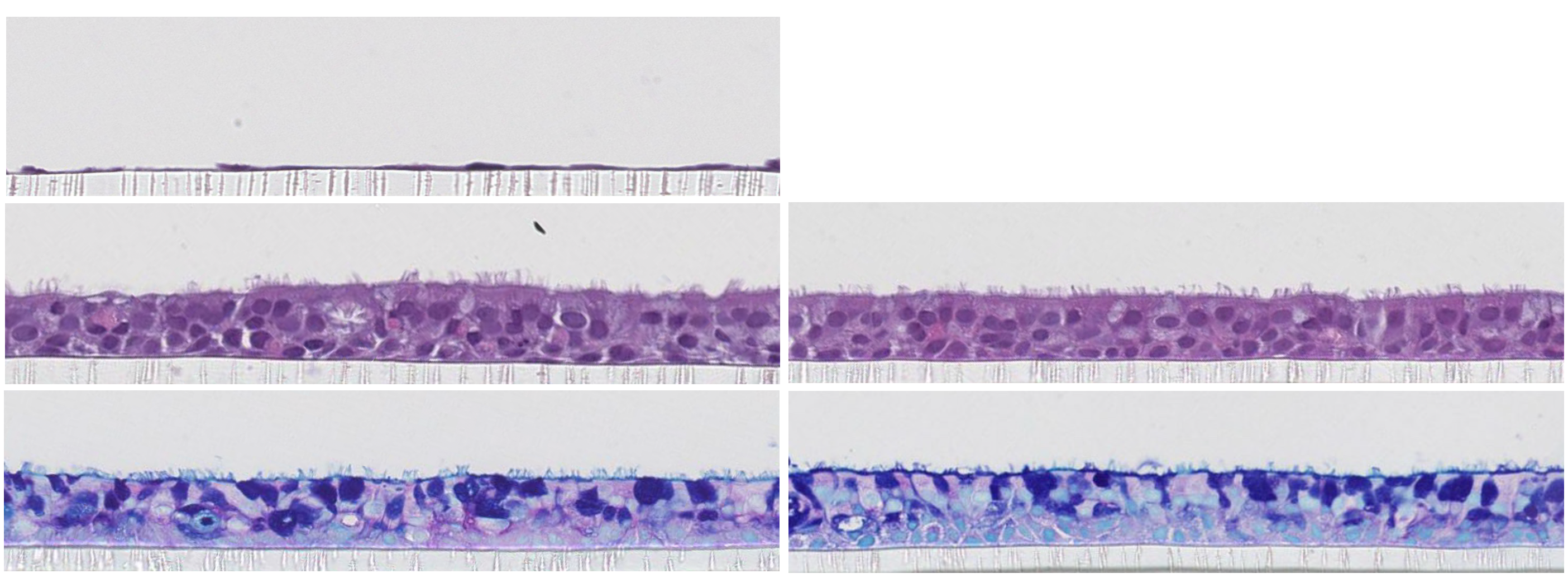

Supplement: Supplementary file 5 — Source data Fig. 2 [file 44318_2025_546_MOESM5_ESM.zip › Figure 2 resubmission of data/2A HE.tif]

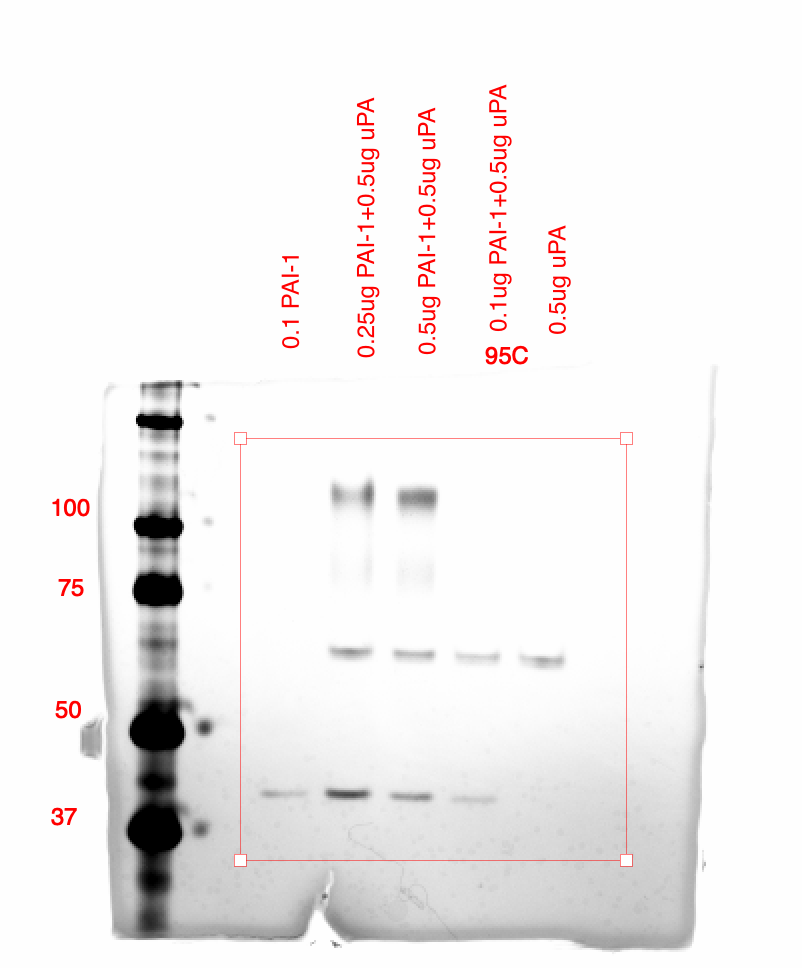

Supplement: Supplementary file 7 — Source data Fig. 4 [file 44318_2025_546_MOESM7_ESM.zip › Figure 4/Fig 4C Blot.png]

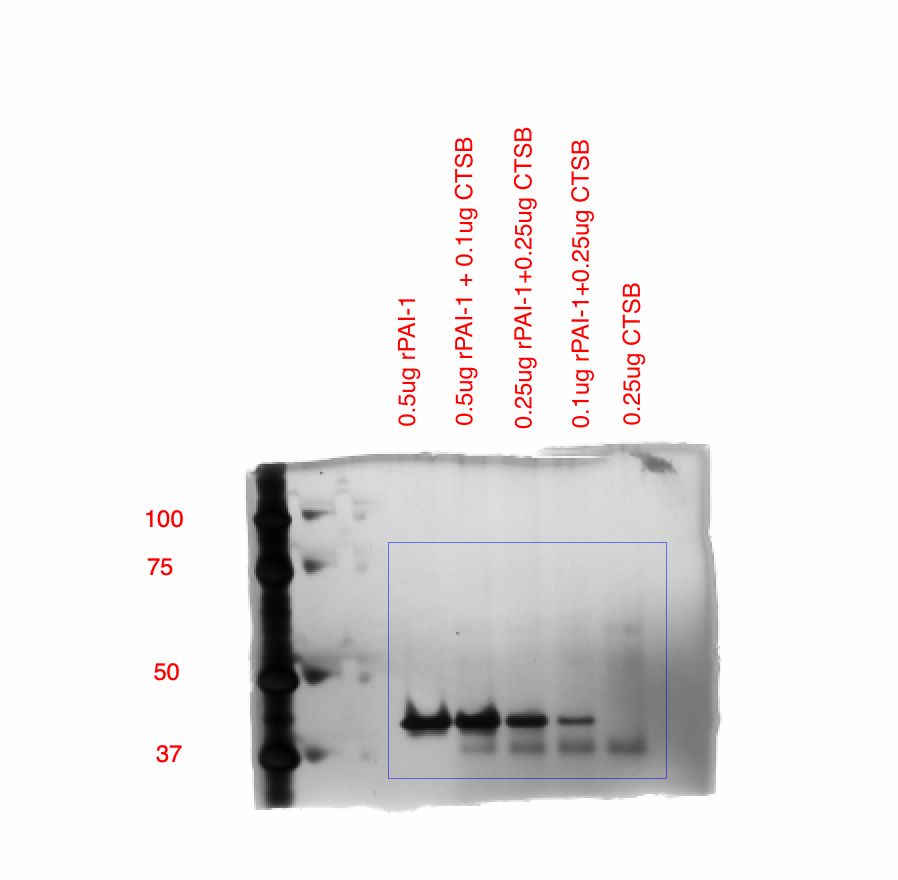

Supplement: Supplementary file 7 — Source data Fig. 4 [file 44318_2025_546_MOESM7_ESM.zip › Figure 4/Fig4G Blot New Panel.png]

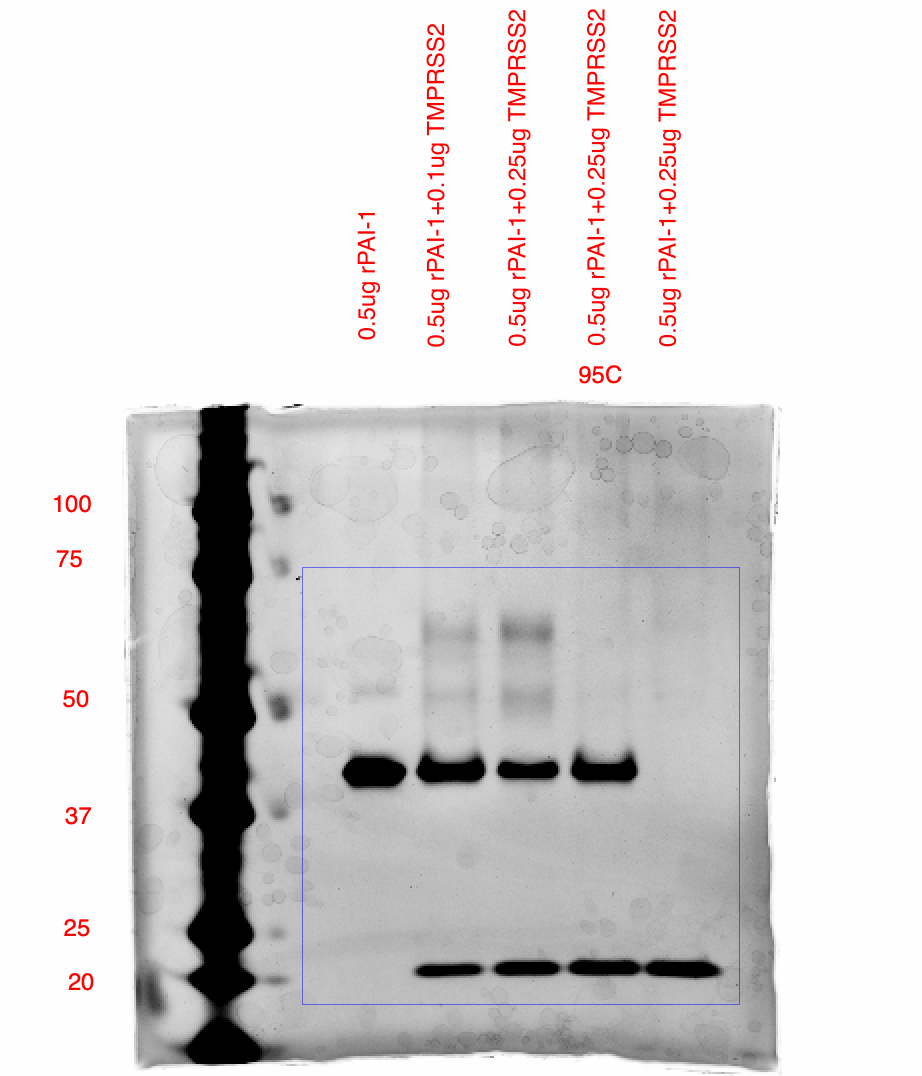

Supplement: Supplementary file 7 — Source data Fig. 4 [file 44318_2025_546_MOESM7_ESM.zip › Figure 4/Fig 4D Blot.png]

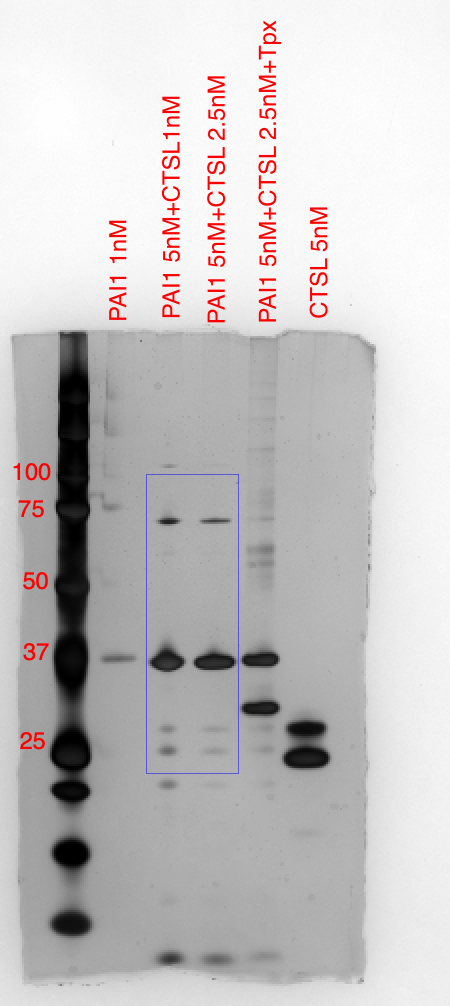

Supplement: Supplementary file 7 — Source data Fig. 4 [file 44318_2025_546_MOESM7_ESM.zip › Figure 4/Fig 4F pH6.5.png]

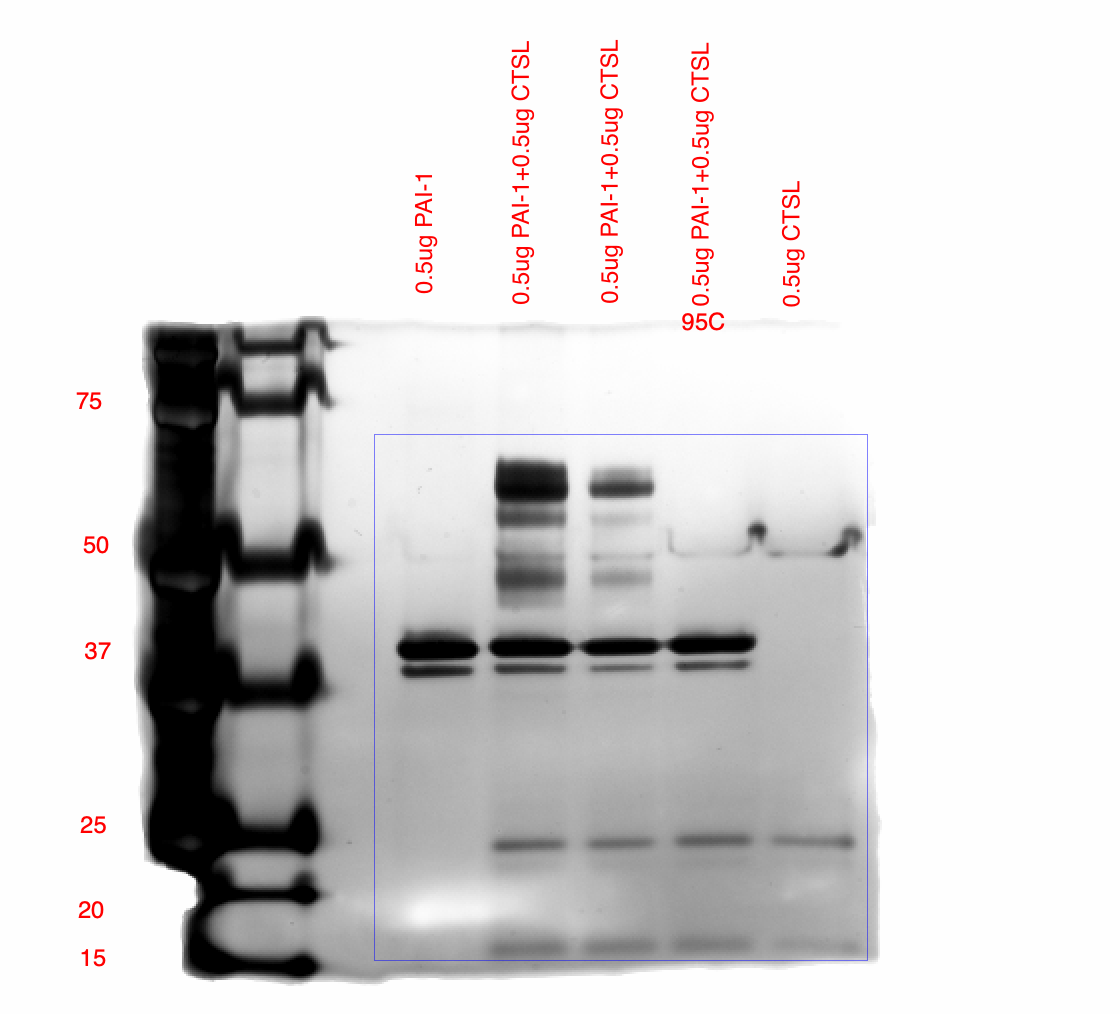

Supplement: Supplementary file 7 — Source data Fig. 4 [file 44318_2025_546_MOESM7_ESM.zip › Figure 4/Fig 4E Blot 1.png]

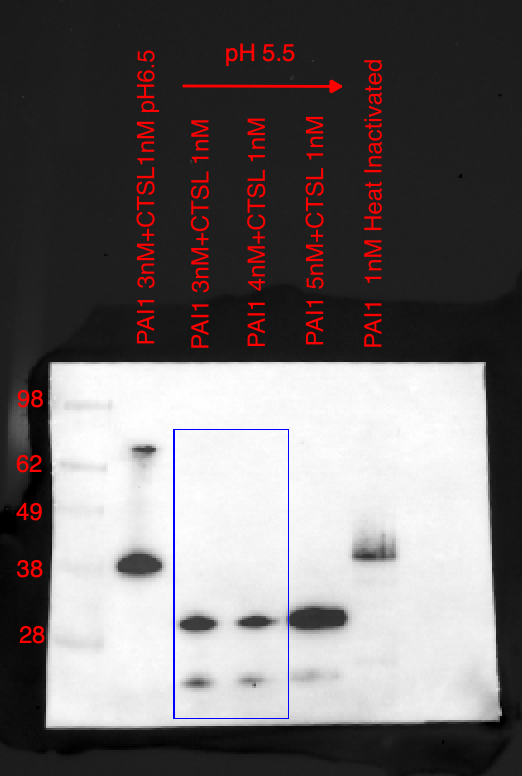

Supplement: Supplementary file 7 — Source data Fig. 4 [file 44318_2025_546_MOESM7_ESM.zip › Figure 4/Fig 4F pH5.5.png]

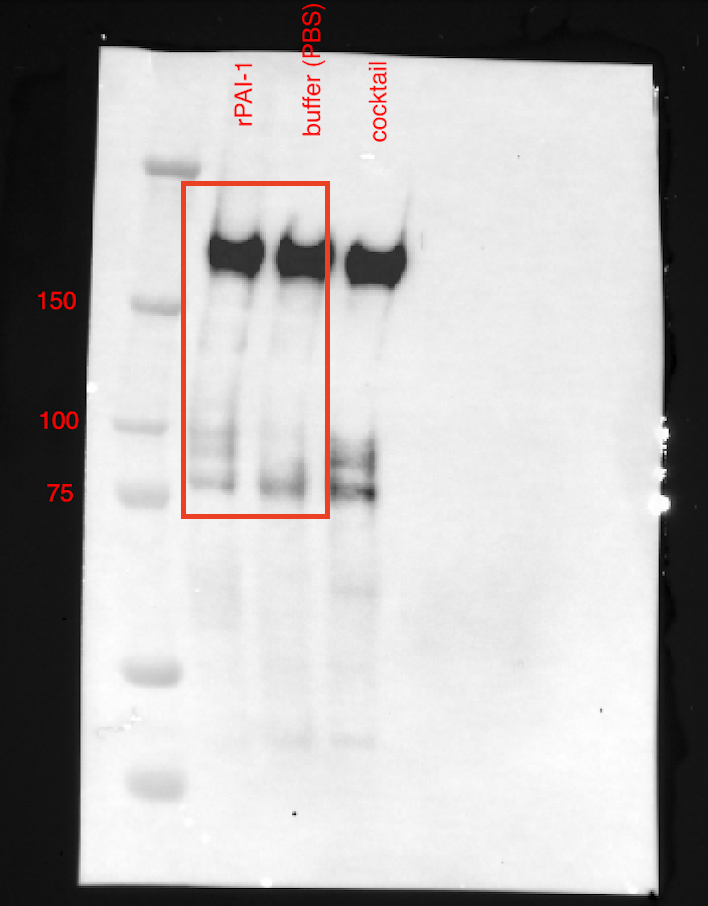

Supplement: Supplementary file 8 — Source data Fig. 5 [file 44318_2025_546_MOESM8_ESM.zip › Figure 5/5G SARS-CoV-2 Spike Blot.png]

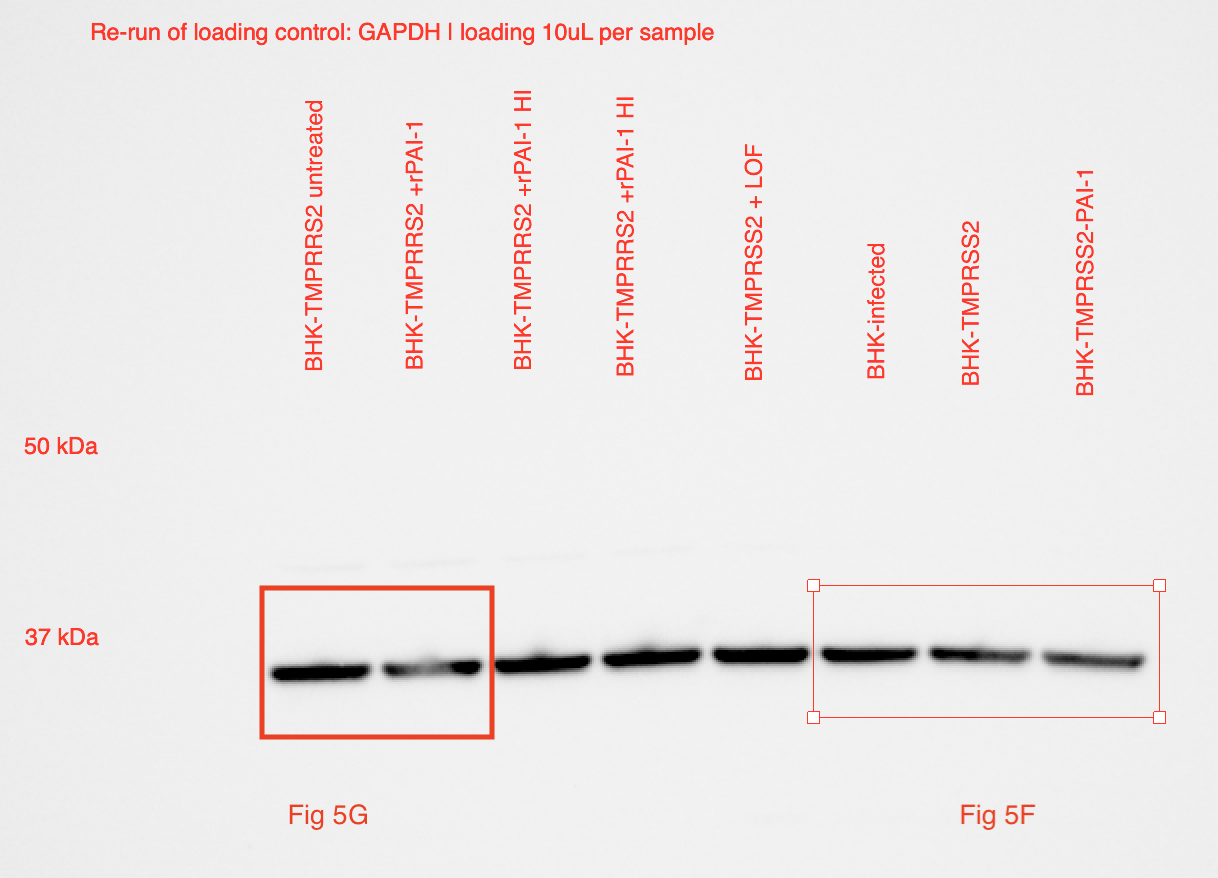

Supplement: Supplementary file 8 — Source data Fig. 5 [file 44318_2025_546_MOESM8_ESM.zip › Figure 5/5F & 5G Blot GAPDH.png]

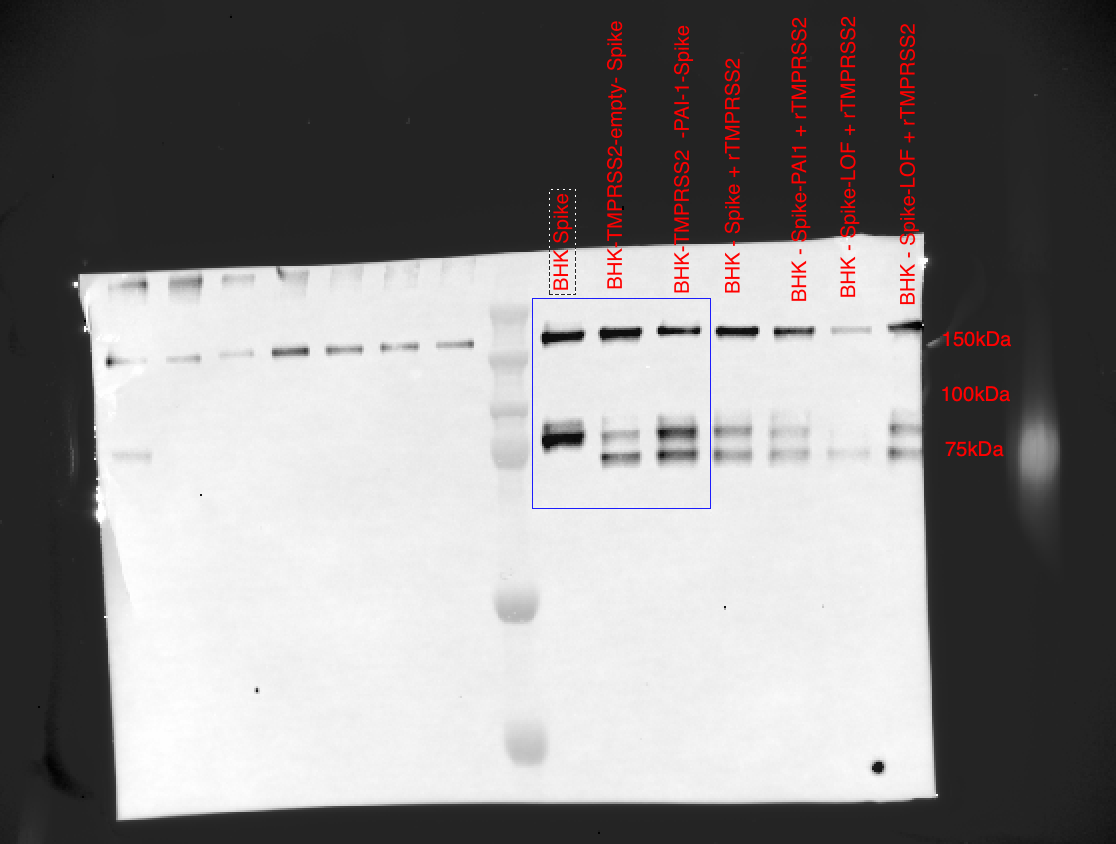

Supplement: Supplementary file 8 — Source data Fig. 5 [file 44318_2025_546_MOESM8_ESM.zip › Figure 5/5F SARS-CoV-2 Spike Blot.png]

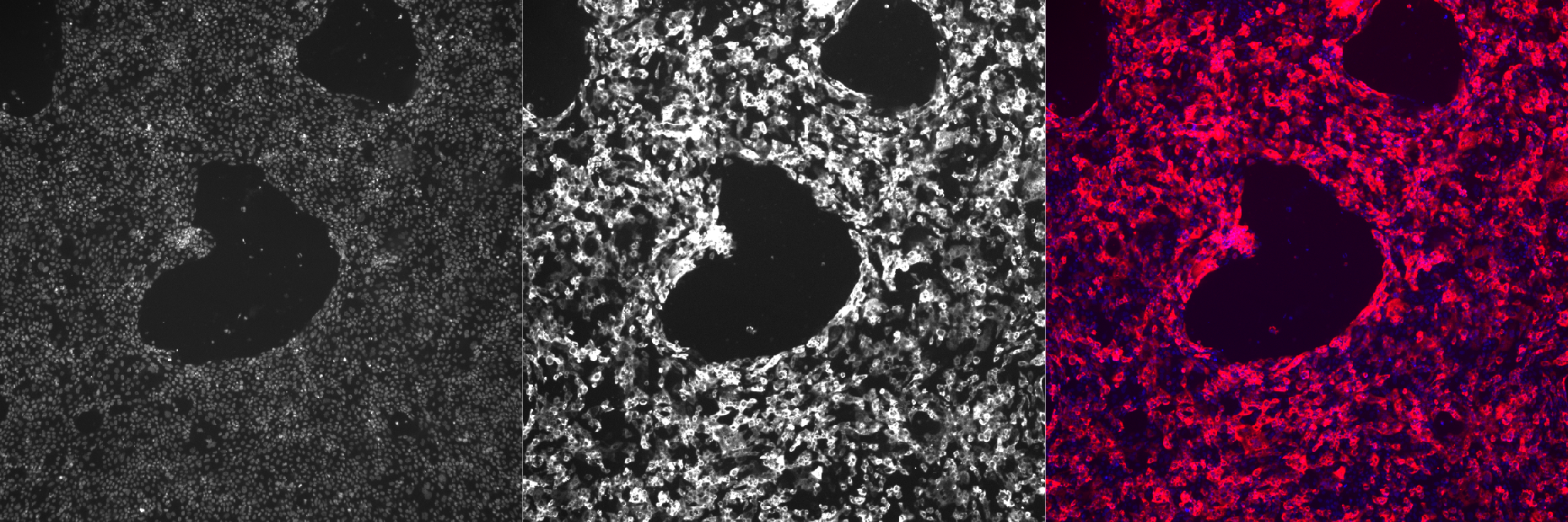

Supplement: Supplementary file 8 — Source data Fig. 5 [file 44318_2025_546_MOESM8_ESM.zip › Figure 5/5D/BAI1_antiPAI1.bmp]

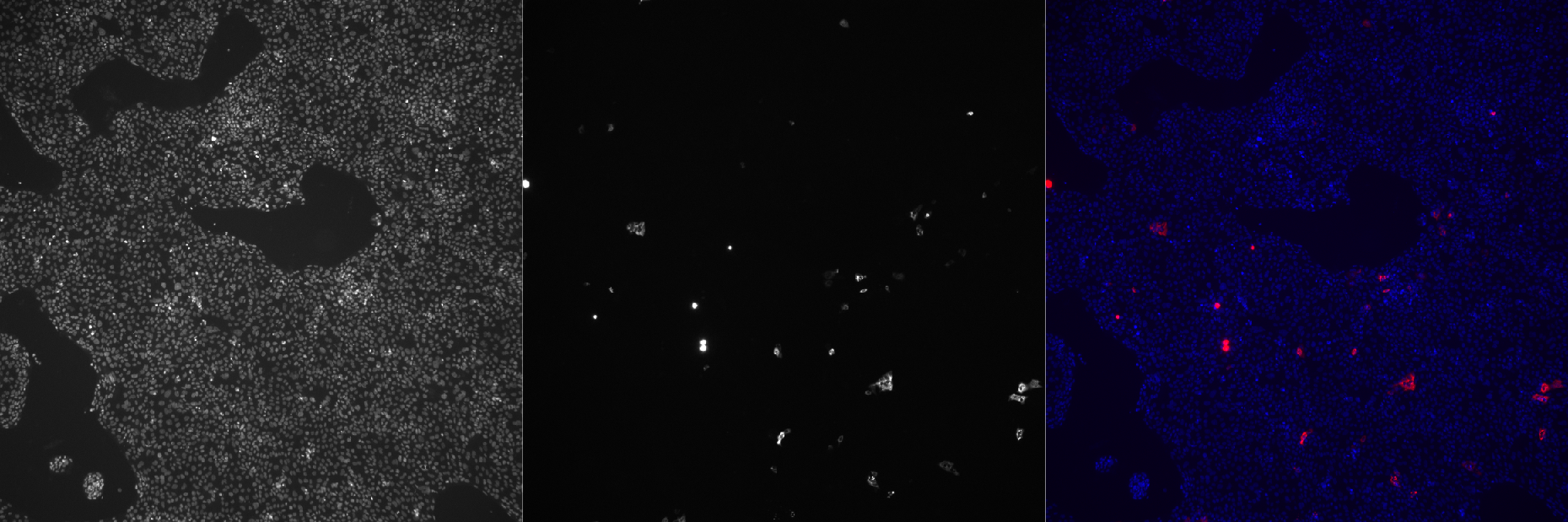

Supplement: Supplementary file 8 — Source data Fig. 5 [file 44318_2025_546_MOESM8_ESM.zip › Figure 5/5D/BA1_rPAI.bmp]

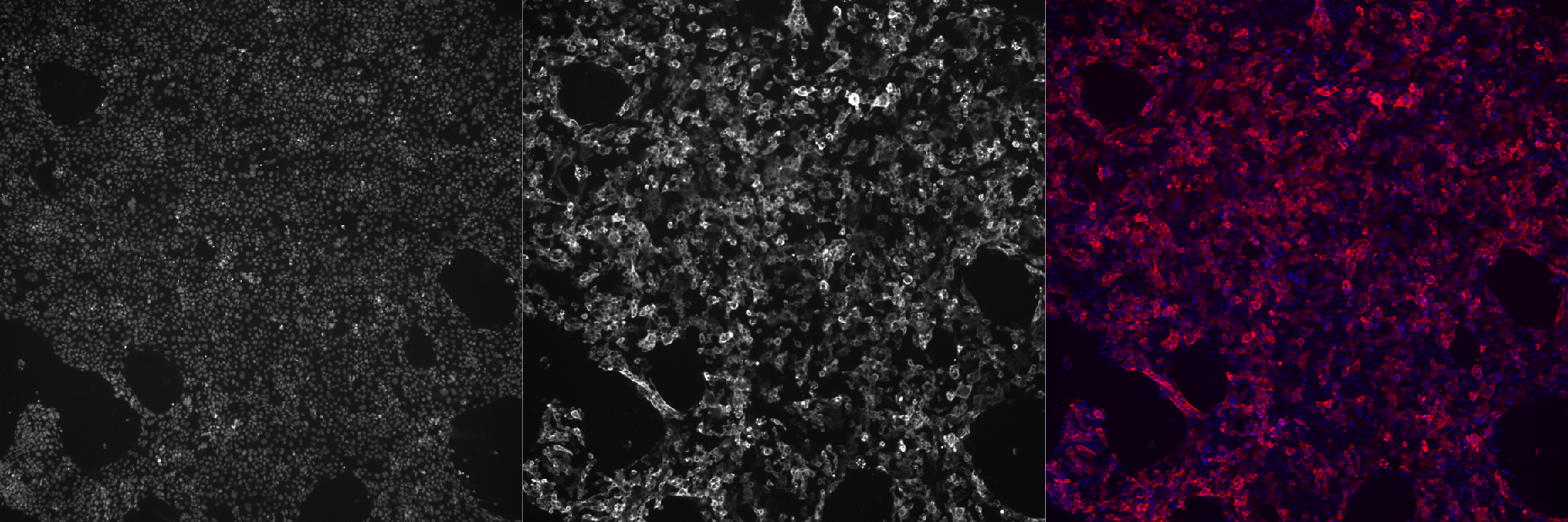

Supplement: Supplementary file 8 — Source data Fig. 5 [file 44318_2025_546_MOESM8_ESM.zip › Figure 5/5D/BA1_buffer (1).bmp]

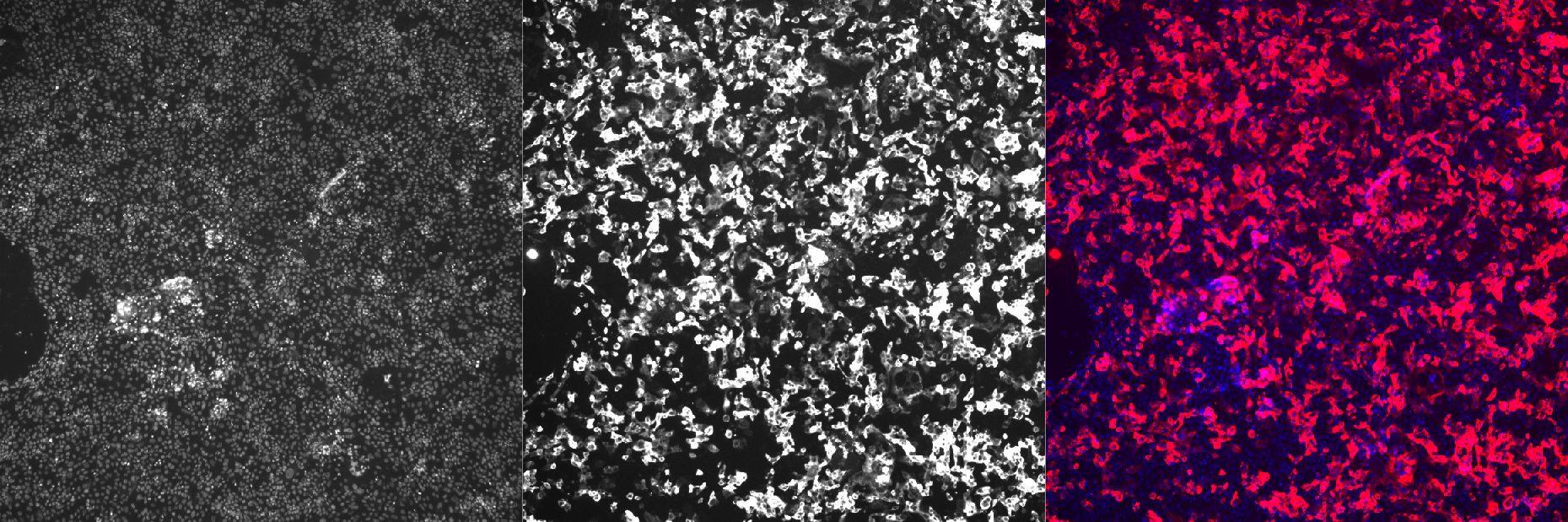

Supplement: Supplementary file 8 — Source data Fig. 5 [file 44318_2025_546_MOESM8_ESM.zip › Figure 5/5D/BAI1_IgG.bmp]

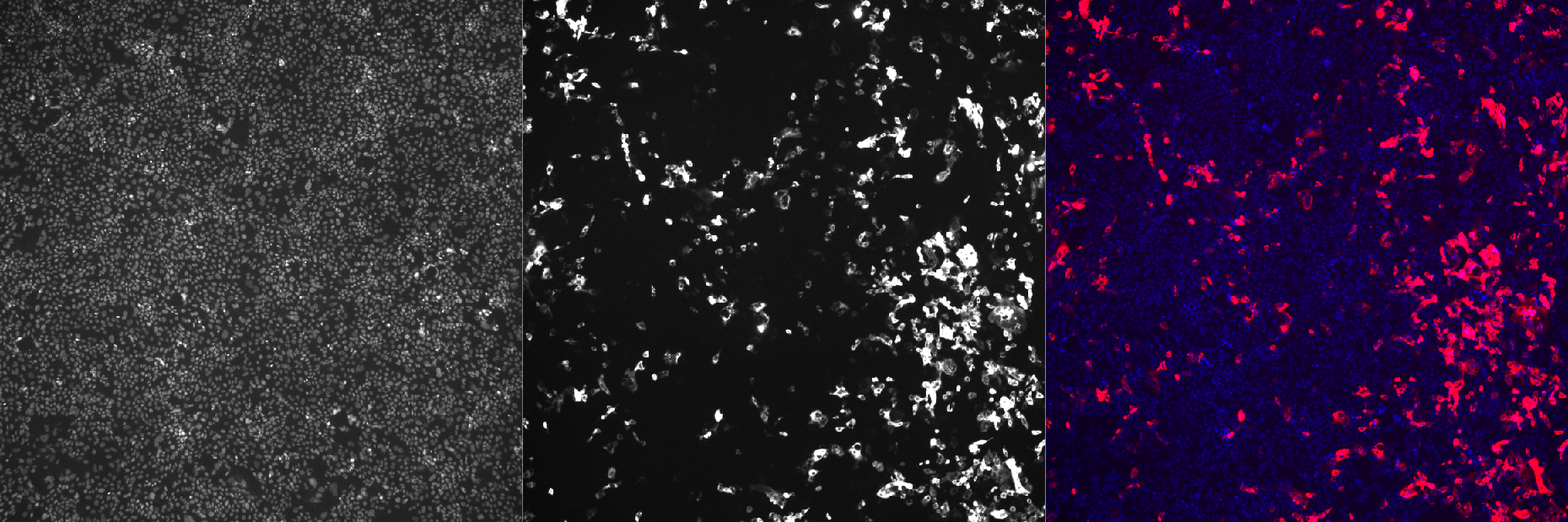

Supplement: Supplementary file 8 — Source data Fig. 5 [file 44318_2025_546_MOESM8_ESM.zip › Figure 5/5B/WA1_IgG.bmp]

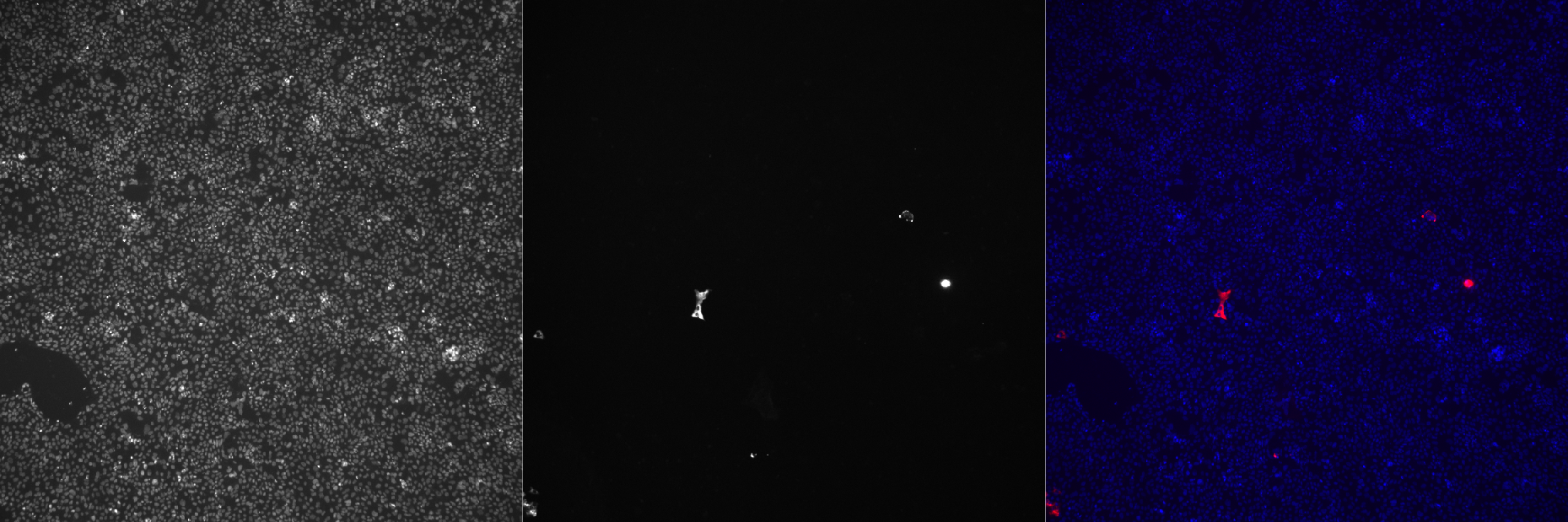

Supplement: Supplementary file 8 — Source data Fig. 5 [file 44318_2025_546_MOESM8_ESM.zip › Figure 5/5B/WA_rPAI1.bmp]

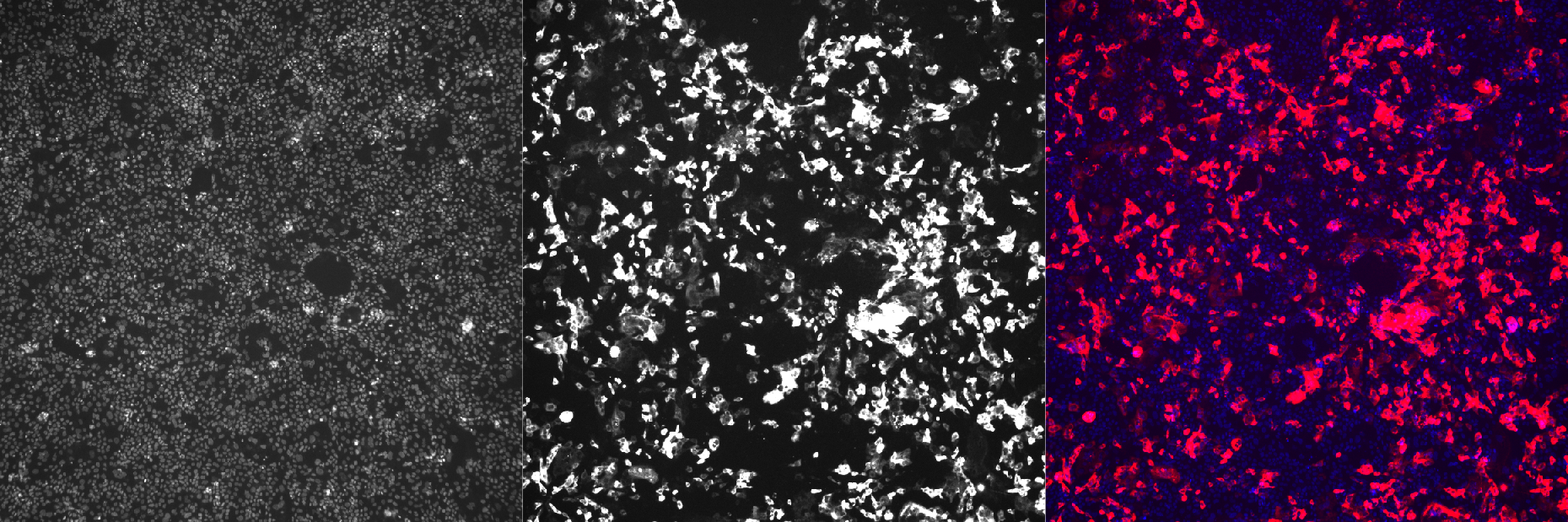

Supplement: Supplementary file 8 — Source data Fig. 5 [file 44318_2025_546_MOESM8_ESM.zip › Figure 5/5B/WA1_antiPAI.bmp]

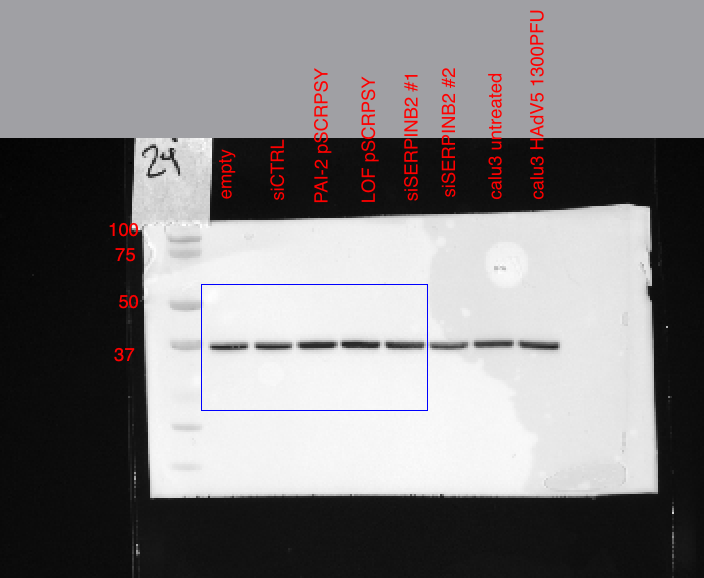

Supplement: Supplementary file 9 — Source data Fig. 6 [file 44318_2025_546_MOESM9_ESM.zip › Figure 6/6L GAPDH Blot.png]

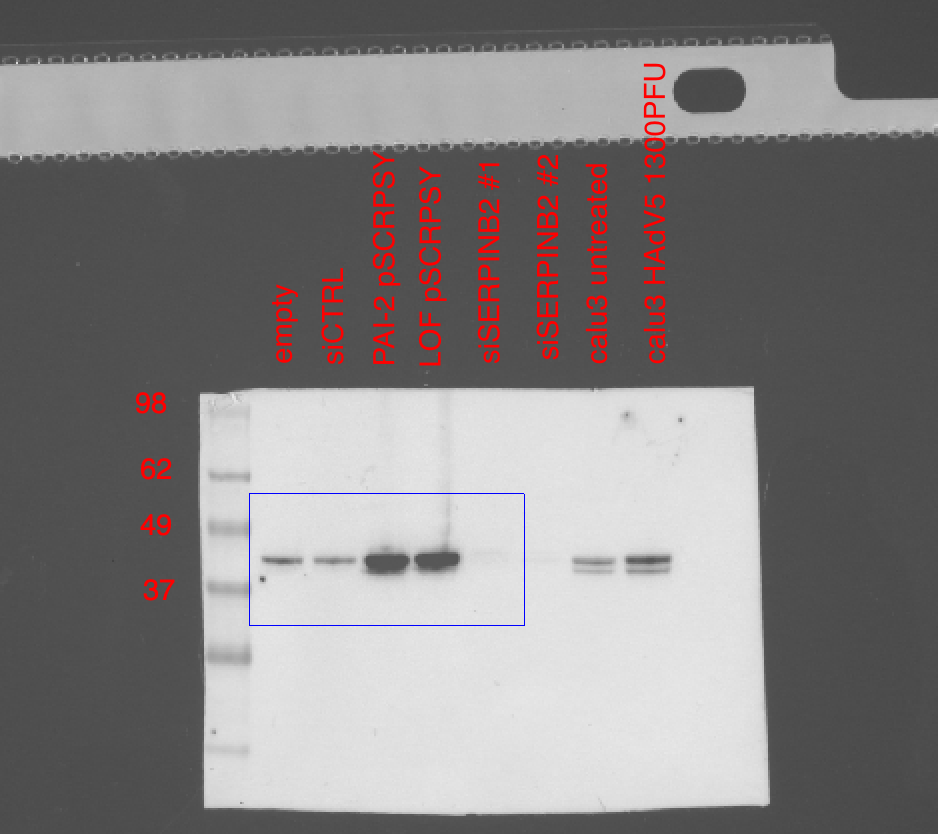

Supplement: Supplementary file 9 — Source data Fig. 6 [file 44318_2025_546_MOESM9_ESM.zip › Figure 6/6L PAI-2 Blot.png]

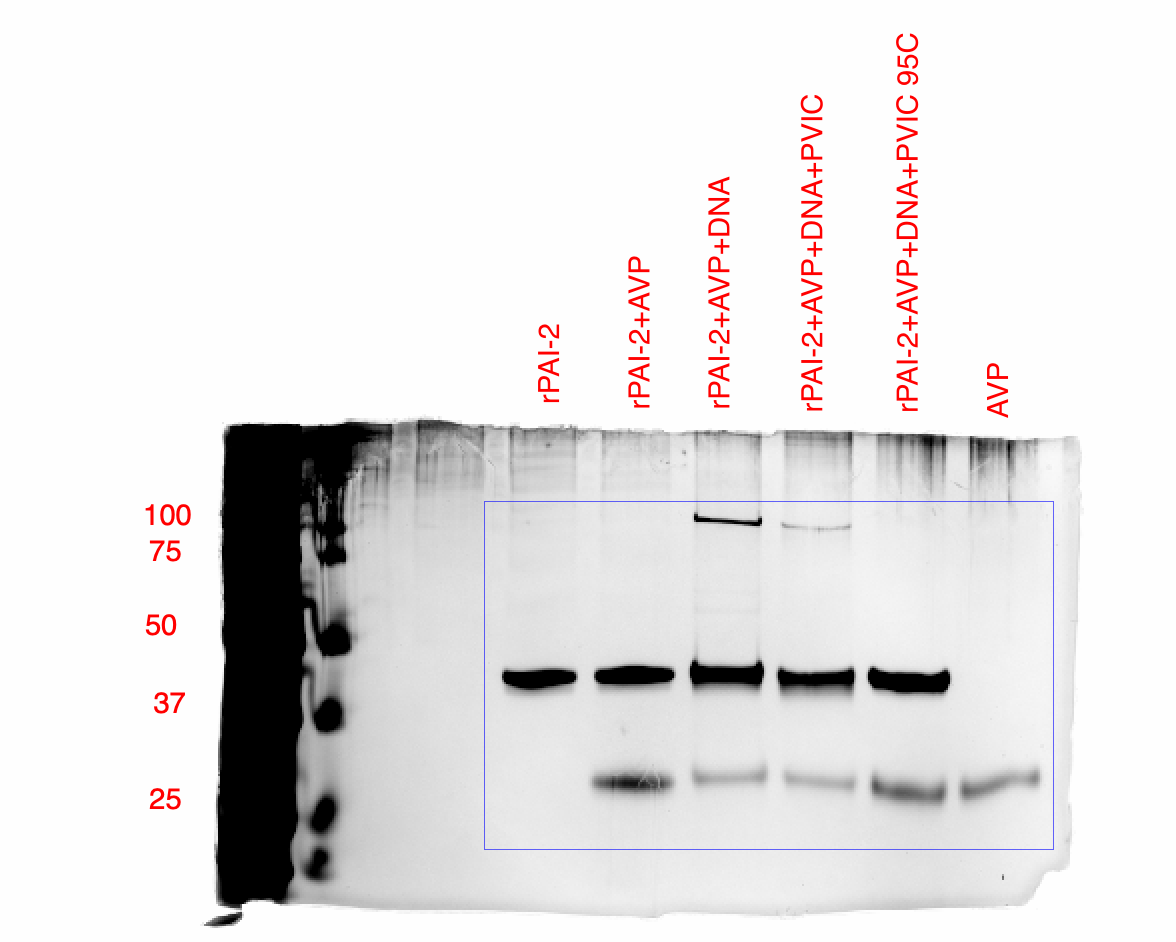

Supplement: Supplementary file 9 — Source data Fig. 6 [file 44318_2025_546_MOESM9_ESM.zip › Figure 6/6G Blot.png]
