# Supplementary material for: Host cell and viral protease targets of human SERPINs identified by in silico docking
Source: EMBO J. 2025 Sep 8;44(20):5755–84. doi: 10.1038/s44318-025-00546-6 (PMC12528359; doi:10.1038/s44318-025-00546-6)
Supplement: Supplementary file 8 — Source data Fig. 5 [file 44318_2025_546_MOESM8_ESM.zip › Figure 5/5B/5B README.rtf]

Microscopy Images for SARS-CoV-2 WA-1 treated with different conditionsLeft panel: DAPI (405nm)Center panel: SARS-CoV-2 (647nm)Left Panel: Merge
