# Supplementary material for: Host cell and viral protease targets of human SERPINs identified by in silico docking
Source: EMBO J. 2025 Sep 8;44(20):5755–84. doi: 10.1038/s44318-025-00546-6 (PMC12528359; doi:10.1038/s44318-025-00546-6)
Supplement: Supplementary file 10 — Table EV1 [file 44318_2025_546_MOESM10_ESM.docx]

**Table EV1. Individual P-values of up- and, downregulated SERPINs in HAEC.**

Individual p-values from log 2 fold change compared to mock in infected HAEC with IAV, HAdV-5C, ReoV, ReoV, PIV3, SARS-CoV-2 or treated with Interferon-beta, obtained by 2-way ANOVA and Fisher’s LSD comparison for data, ashown in **Figure 2b.** n=3.

| ***IFITM3*** | **RhV** | **ReoV** | **IAV** | **PIV3** | **AdV** | **SARS2** |
| --- | --- | --- | --- | --- | --- | --- |
| 0 vs. 24 | 0.4226 | 0.3786 | 0.1338 | 0.4226 | 0.0244* | 0.4956 |
| 0 vs. 72 | 0.0038** | 0.1301 | 0.1259 | 0.4226 | 0.1077 | 0.3091 |
|  |  |  |  |  |  |  |
| ***SERPINA1*** | **RhV** | **ReoV** | **IAV** | **PIV3** | **AdV** | **SARS2** |
| 0 vs. 24 | 0.8353 | 0.8858 | 0.3628 | 0.2769 | 0.1274 | 0.0111* |
| 0 vs. 72 | 0.2388 | 0.4342 | 0.4975 | 0.0705 | 0.0295 | 0.489 |
|  |  |  |  |  |  |  |
| ***SERPINA4*** | **RhV** | **ReoV** | **IAV** | **PIV3** | **AdV** | **SARS2** |
| 0 vs. 24 | 0.0498* | 0.2149 | 0.7562 | 0.8075 | 0.2436 | 0.0108* |
| 0 vs. 72 | 0.2139 | 0.0238 | 0.8467 | 0.369 | 0.9655 | 0.1553 |
|  |  |  |  |  |  |  |
| ***SERPINA5*** | **RhV** | **ReoV** | **IAV** | **PIV3** | **AdV** | **SARS2** |
| 0 vs. 24 | 0.4226 | 0.0106* | 0.1841 | 0.0958 | 0.2777 | 0.248 |
| 0 vs. 72 | 0.017* | 0.0878 | 0.1855 | 0.2744 | 0.0437* | 0.4365 |
|  |  |  |  |  |  |  |
| ***SERPINA6*** | **RhV** | **ReoV** | **IAV** | **PIV3** | **AdV** | **SARS2** |
| 0 vs. 24 | 0.1728 | 0.5571 | 0.2223 | 0.1999 | 0.0409* | 0.0012** |
| 0 vs. 72 | 0.3355 | 0.1499 | 0.2854 | 0.9958 | 0.4916 | 0.0895 |
|  |  |  |  |  |  |  |
| ***SERPINA7*** | **RhV** | **ReoV** | **IAV** | **PIV3** | **AdV** | **SARS2** |
| 0 vs. 24 | n.d. | n.d. | n.d. | n.d. | n.d. | 0.1847 |
| 0 vs. 72 | n.d. | n.d. | n.d. | n.d. | n.d. | 0.4226 |
|  |  |  |  |  |  |  |
| ***SERPINA9*** | **RhV** | **ReoV** | **IAV** | **PIV3** | **AdV** | **SARS2** |
| 0 vs. 24 | 0.0699 | 0.1622 | 0.185 | 0.0967 | 0.187 | 0.0144* |
| 0 vs. 72 | 0.5239 | 0.0466 | 0.2329 | 0.0018 | 0.9988 | 0.0438* |
|  |  |  |  |  |  |  |
| ***SERPINA10*** | **RhV** | **ReoV** | **IAV** | **PIV3** | **AdV** | **SARS2** |
| 0 vs. 24 | n.d. | n.d. | 0.1991 | 0.1835 | n.d. | 0.0058** |
| 0 vs. 72 | n.d. | n.d. | 0.2254 | 0.1835 | n.d. | 0.6546 |
|  |  |  |  |  |  |  |
| ***SERPINA11*** | **RhV** | **ReoV** | **IAV** | **PIV3** | **AdV** | **SARS2** |
| 0 vs. 24 | 0.0587 | 0.0175* | 0.269 | 0.1245 | 0.1777 | 0.4226 |
| 0 vs. 72 | 0.0853 | 0.0601 | 0.4364 | 0.1304 | 0.2302 | 0.4226 |
|  |  |  |  |  |  |  |
| ***SERPINA12*** | **RhV** | **ReoV** | **IAV** | **PIV3** | **AdV** | **SARS2** |
| 0 vs. 24 | 0.3837 | 0.1584 | 0.4226 | 0.1041 | 0.1646 | 0.2532 |
| 0 vs. 72 | 0.1575 | 0.0654 | 0.4226 | 0.4979 | 0.0941 | 0.189 |
|  |  |  |  |  |  |  |
| ***SERPINB1*** | **RhV** | **ReoV** | **IAV** | **PIV3** | **AdV** | **SARS2** |
| 0 vs. 24 | 0.1606 | 0.9094 | 0.4226 | 0.1835 | 0.0287* | n.d. |
| 0 vs. 72 | 0.2564 | 0.441 | 0.4226 | 0.6606 | 0.1144 | n.d. |
|  |  |  |  |  |  |  |
| ***SERPINB2*** | **RhV** | **ReoV** | **IAV** | **PIV3** | **AdV** | **SARS2** |
| 0 vs. 24 | 0.1483 | 0.7501 | 0.1094 | 0.0164* | 0.0805 | 0.0155* |
| 0 vs. 72 | 0.1687 | 0.3408 | 0.0764 | 0.0646 | 0.0876 | n.d. |
|  |  |  |  |  |  |  |
| ***SERPINB3*** | **RhV** | **ReoV** | **IAV** | **PIV3** | **AdV** | **SARS2** |
| 0 vs. 24 | 0.0072* | 0.0483* | 0.1126 | 0.0366* | 0.0195* | 0.1898 |
| 0 vs. 72 | 0.0392* | 0.299 | 0.2848 | 0.659 | 0.0756 | 0.189 |
|  |  |  |  |  |  |  |
| ***SERPINB4*** | **RhV** | **ReoV** | **IAV** | **PIV3** | **AdV** | **SARS2** |
| 0 vs. 24 | 0.0137* | 0.2753 | 0.0955 | 0.0089* | 0.0981 | 0.1886 |
| 0 vs. 72 | 0.0499 | 0.1655 | 0.8861 | 0.0721 | 0.1783 | 0.1932 |
|  |  |  |  |  |  |  |
| ***SERPINB5*** | **RhV** | **ReoV** | **IAV** | **PIV3** | **AdV** | **SARS2** |
| 0 vs. 24 | 0.1303 | 0.0629 | 0.1536 | 0.0591 | 0.0019** | 0.021* |
| 0 vs. 72 | 0.1913 | 0.3482 | 0.2087 | 0.0721 | 0.0943 | 0.1678 |
|  |  |  |  |  |  |  |
| ***SERPINB6*** | **RhV** | **ReoV** | **IAV** | **PIV3** | **AdV** | **SARS2** |
| 0 vs. 24 | 0.342 | 0.6209 | 0.1813 | 0.4088 | 0.0122* | 0.0038** |
| 0 vs. 72 | 0.3263 | 0.2205 | 0.36 | 0.3963 | 0.7787 | 0.0171* |
|  |  |  |  |  |  |  |
| ***SERPINB8*** | **RhV** | **ReoV** | **IAV** | **PIV3** | **AdV** | **SARS2** |
| 0 vs. 24 | 0.0172* | 0.3256 | 0.0287* | 0.0129* | 0.0363* | 0.0061* |
| 0 vs. 72 | 0.144 | 0.0813 | 0.0015** | 0.3702 | 0.1327 | 0.1403 |
|  |  |  |  |  |  |  |
| ***SERPINB9*** | **RhV** | **ReoV** | **IAV** | **PIV3** | **AdV** | **SARS2** |
| 0 vs. 24 | 0.0142* | 0.3356 | 0.1347 | 0.0125* | 0.1647 | 0.0002*** |
| 0 vs. 72 | 0.0995 | 0.162 | 0.2008 | 0.3445 | 0.1683 | 0.0677 |
|  |  |  |  |  |  |  |
| ***SERPINB10*** | **RhV** | **ReoV** | **IAV** | **PIV3** | **AdV** | **SARS2** |
| 0 vs. 24 | 0.0056* | 0.7631 | 0.1687 | 0.0246* | 0.1604 | 0.0128* |
| 0 vs. 72 | 0.2079 | 0.1041 | 0.0376* | 0.3667 | 0.459 | 0.2474 |
|  |  |  |  |  |  |  |
| ***SERPINB11*** | **RhV** | **ReoV** | **IAV** | **PIV3** | **AdV** | **SARS2** |
| 0 vs. 24 | 0.0225* | <0.0001**** | 0.8606 | 0.0015** | n.d. | 0.1048 |
| 0 vs. 72 | 0.599 | n.d. | 0.0661 | 0.4043 | n.d. | 0.836 |
|  |  |  |  |  |  |  |
| ***SERPINB13*** | **RhV** | **ReoV** | **IAV** | **PIV3** | **AdV** | **SARS2** |
| 0 vs. 24 | 0.4896 | 0.4145 | 0.1173 | 0.0904 | 0.0844 | 0.4226 |
| 0 vs. 72 | 0.3111 | 0.1675 | 0.1304 | 0.4079 | 0.2223 | 0.4527 |
|  |  |  |  |  |  |  |
| ***SERPINC1*** | **RhV** | **ReoV** | **IAV** | **PIV3** | **AdV** | **SARS2** |
| 0 vs. 24 | 0.0359* | <0.0001**** | n.d. | n.d. | n.d. | 0.1859 |
| 0 vs. 72 | n.d. | <0.0001**** | n.d. | 0.0911 | 0.4226 | 0.8216 |
|  |  |  |  |  |  |  |
| ***SERPIND1*** | **RhV** | **ReoV** | **IAV** | **PIV3** | **AdV** | **SARS2** |
| 0 vs. 24 | 0.678 | 0.0612 | 0.1359 | 0.6402 | 0.0706 | <0.0001**** |
| 0 vs. 72 | 0.231 | 0.0095* | 0.9182 | 0.261 | 0.1026 | 0.4226 |
|  |  |  |  |  |  |  |
| ***SERPINE1*** | **RhV** | **ReoV** | **IAV** | **PIV3** | **AdV** | **SARS2** |
| 0 vs. 24 | 0.0078* | 0.3258 | 0.5016 | 0.0074* | 0.0655 | 0.1835 |
| 0 vs. 72 | 0.1645 | 0.1703 | 0.371 | 0.3724 | 0.1067 | 0.477 |
|  |  |  |  |  |  |  |
| ***SERPINE2*** | **RhV** | **ReoV** | **IAV** | **PIV3** | **AdV** | **SARS2** |
| 0 vs. 24 | <0.0001**** | n.d. | n.d. | n.d. | n.d. | n.d. |
| 0 vs. 72 | n.d. | 0.0651 | 0.4226 | 0.1835 | n.d. | n.d. |
|  |  |  |  |  |  |  |
| ***SERPINF1*** | **RhV** | **ReoV** | **IAV** | **PIV3** | **AdV** | **SARS2** |
| 0 vs. 24 | 0.3805 | 0.6196 | 0.5878 | 0.8236 | 0.0003*** | 0.6005 |
| 0 vs. 72 | 0.4384 | 0.1881 | 0.6506 | 0.4269 | 0.1623 | n.d. |
|  |  |  |  |  |  |  |
| ***SERPINF2*** | **RhV** | **ReoV** | **IAV** | **PIV3** | **AdV** | **SARS2** |
| 0 vs. 24 | 0.043* | 0.8708 | 0.3393 | 0.9403 | 0.0926 | 0.0002*** |
| 0 vs. 72 | 0.4292 | 0.265 | 0.3421 | 0.4052 | 0.1127 | 0.4226 |
|  |  |  |  |  |  |  |
| ***SERPING1*** | **RhV** | **ReoV** | **IAV** | **PIV3** | **AdV** | **SARS2** |
| 0 vs. 24 | 0.0462* | 0.1801 | 0.0021** | 0.0171* | 0.0824 | 0.1926 |
| 0 vs. 72 | 0.0608 | 0.1275 | 0.039* | 0.3118 | 0.0003*** | 0.4228 |
|  |  |  |  |  |  |  |
| ***SERPINI2*** | **RhV** | **ReoV** | **IAV** | **PIV3** | **AdV** | **SARS2** |
| 0 vs. 24 | 0.0378* | 0.7231 | 0.2212 | 0.3661 | 0.3201 | 0.1835 |
| 0 vs. 72 | 0.848 | 0.3219 | 0.1529 | 0.409 | 0.3925 | 0.4226 |

| ***IFITM3*** | **IFN beta** |
| --- | --- |
| 0 vs. 6 | 0.0239* |
| 0 vs. 12 | 0.0074** |
| 0 vs. 24 | 0.0347** |
|  |  |
| ***SERPINA1*** | |
| 0 vs. 6 | 0.1759 |
| 0 vs. 12 | 0.7406 |
| 0 vs. 24 | 0.6521 |
|  |  |
| ***SERPINA4*** | |
| 0 vs. 6 | n.d. |
| 0 vs. 12 | n.d. |
| 0 vs. 24 | n.d. |
|  |  |
| ***SERPINA5*** | |
| 0 vs. 6 | n.d. |
| 0 vs. 12 | n.d. |
| 0 vs. 24 | n.d. |
|  |  |
| ***SERPINA6*** | |
| 0 vs. 6 | 0.1357 |
| 0 vs. 12 | 0.7537 |
| 0 vs. 24 | 0.5018 |
|  |  |
| ***SERPINA7*** | |
| 0 vs. 6 | n.d. |
| 0 vs. 12 | n.d. |
| 0 vs. 24 | n.d. |
|  |  |
| ***SERPINA9*** | |
| 0 vs. 6 | n.d. |
| 0 vs. 12 | n.d. |
| 0 vs. 24 | n.d. |
|  |  |
| ***SERPINA10*** | |
| 0 vs. 6 | n.d. |
| 0 vs. 12 | n.d. |
| 0 vs. 24 | n.d. |
|  |  |
| ***SERPINA11*** | |
| 0 vs. 6 | 0.4426 |
| 0 vs. 12 | 0.9307 |
| 0 vs. 24 | 0.8225 |
|  |  |
| ***SERPINA12*** | |
| 0 vs. 6 | 0.7296 |
| 0 vs. 12 | 0.4618 |
| 0 vs. 24 | 0.6405 |
|  |  |
| ***SERPINB1*** | |
| 0 vs. 6 | 0.0021** |
| 0 vs. 12 | 0.9187 |
| 0 vs. 24 | 0.1561 |
|  |  |
| ***SERPINB2*** | |
| 0 vs. 6 | 0.1384 |
| 0 vs. 12 | 0.0499* |
| 0 vs. 24 | 0.0971 |
|  |  |
| ***SERPINB3*** | |
| 0 vs. 6 | 0.3437 |
| 0 vs. 12 | 0.7509 |
| 0 vs. 24 | 0.372 |
|  |  |
| ***SERPINB4*** | |
| 0 vs. 6 | 0.4126 |
| 0 vs. 12 | 0.6494 |
| 0 vs. 24 | 0.8268 |
|  |  |
| ***SERPINB5*** | |
| 0 vs. 6 | 0.2065 |
| 0 vs. 12 | 0.9138 |
| 0 vs. 24 | 0.0758 |
|  |  |
| ***SERPINB6*** | |
| 0 vs. 6 | 0.0614 |
| 0 vs. 12 | 0.9887 |
| 0 vs. 24 | 0.1748 |
|  |  |
| ***SERPINB8*** | |
| 0 vs. 6 | 0.0117* |
| 0 vs. 12 | 0.2194 |
| 0 vs. 24 | 0.0767 |
|  |  |
| ***SERPINB9*** | |
| 0 vs. 6 | 0.02* |
| 0 vs. 12 | 0.0939 |
| 0 vs. 24 | 0.0237 |
|  |  |
| ***SERPINB10*** | |
| 0 vs. 6 | 0.0356* |
| 0 vs. 12 | 0.1378 |
| 0 vs. 24 | 0.0547 |
|  |  |
| ***SERPINB11*** | |
| 0 vs. 6 | 0.1571 |
| 0 vs. 12 | 0.0233* |
| 0 vs. 24 | 0.0866 |
|  |  |
| ***SERPINB13*** | |
| 0 vs. 6 | 0.076 |
| 0 vs. 12 | 0.2479 |
| 0 vs. 24 | 0.0588 |
|  |  |
| ***SERPINC1*** | |
| 0 vs. 6 | 0.8213 |
| 0 vs. 12 | 0.2202 |
| 0 vs. 24 | 0.1504 |
|  |  |
| ***SERPIND1*** | |
| 0 vs. 6 | 0.6026 |
| 0 vs. 12 | 0.6272 |
| 0 vs. 24 | 0.67 |
|  |  |
| ***SERPINE1*** | |
| 0 vs. 6 | 0.0356* |
| 0 vs. 12 | 0.338 |
| 0 vs. 24 | 0.2368 |
|  |  |
| ***SERPINE2*** | |
| 0 vs. 6 | n.d. |
| 0 vs. 12 | n.d. |
| 0 vs. 24 | n.d. |
|  |  |
| ***SERPINF1*** | |
| 0 vs. 6 | 0.2635 |
| 0 vs. 12 | 0.9645 |
| 0 vs. 24 | 0.4234 |
|  |  |
| ***SERPINF2*** | |
| 0 vs. 6 | 0.6711 |
| 0 vs. 12 | 0.5474 |
| 0 vs. 24 | 0.1595 |
|  |  |
| ***SERPING1*** | |
| 0 vs. 6 | 0.1406 |
| 0 vs. 12 | 0.0236* |
| 0 vs. 24 | 0.0443* |
|  |  |
| ***SERPINI2*** |  |
| 0 vs. 6 | 0.3045 |
| 0 vs. 12 | 0.2983 |
| 0 vs. 24 | 0.3059 |
