# Supplementary material for: Host cell and viral protease targets of human SERPINs identified by in silico docking
Source: EMBO J. 2025 Sep 8;44(20):5755–84. doi: 10.1038/s44318-025-00546-6 (PMC12528359; doi:10.1038/s44318-025-00546-6)
Supplement: Supplementary file 11 — Table EV2 [file 44318_2025_546_MOESM11_ESM.docx]

**Table EV2. Primers used for qPCR of infected or interferon-treated HAEC.**

List of primers used for the qPCR analysis of HAECs infected HAEC with IAV, HAdV-5C, ReoV, ReoV, PIV3, SARS-CoV-2 or treated with Interferon-beta.

| **SERPIN** | **F primer** | **R primer** |
| --- | --- | --- |
| A1 | TCTTCTTCTCCCCAGTGAGCATC | CAGGATTTCATCGTGAGTGTCAGC |
| A4 | GAGGCTAAACTCTTCCACACCAAC | TGACCAAATCCACAATCTTCCC |
| A5 | CAGACACTTTCCCCACCAACTTTAG | CCCTTCGTTTGCTTTGCCAC |
| A6 | CGGAGATGTGCTGGAGGAAATG | TGGACCACCTTTGATGACTTCAG |
| A7 | CCAACCAAATGCCACTCTCTACAAG | TGTTCTTATCTGGGGTCTCCACAG |
| A9 | TGTGCTCCAATCTACTGTGTGTCC | GCAAAGTCGGTGTTGAGGGAATAC |
| A10 | CTCACCCTTTGCTGACCTTAG | GCCCCTTTCATCAACTTCAATCAC |
| A11 | CCACAGAATCACACCCACCATTAC | GGCGAGAAGAAGATGTTTCCG |
| A12 | CCATTTTTCTGGCTGTTCTCCTC | CATCCTTGGACCTCGCTCAAAG |
| B1 | ATGGAGCAGCTGAGCTCAGC | CTAAGGGGAAGAAAATCTCCCC |
| B2 | AAATCCATTCATCCTTCCGCTC | CGCAGACTTCTCACCAAACAGC |
| B3 | CAGCATTAGGGATGGTCCTCTTAG | TCCTGTGGTGTTCTCTGTGACTTG |
| B4 | GCAAATGCTCCAGAAGAAAGTCG | GCCAATAGTCCCATCAGGAAATAGG |
| B5 | GGCAATGTCCTCTTCTCTCCAATC | TGTCACCTTTAGCACCCACTTGAG |
| B6 | CGCCGTAGAGAAGTCCAGAAAAC | CCAGAACCAGCCTTGTCAATGG |
| B8 | TGGAGCCAAGATTCTGTGC | TTTTGTGGTGCCTGATGAAG |
| B9 | GAAACTCACAGCCTGGACCAAG | AAATGCCGAAGCACAGATTCC |
| B10 | TCAGAAAGGACATCAACTCTTGGG | AGGGCGTTCACCAGAATCATCC |
| B11 | ATTCATTCCGAGTTTGGTGTCG | TTTGTCCCGTAGAGCCTGTTGG |
| B13 | TTTACTGTCACATCCGCCCCAG | GAAGAAAATCTGCCGAAGAAGAGG |
| C1 | GGAGAAGAAGGCAACTGAGGATG | AGAAAGTGGTAGCAAAGCGGG |
| D1 | CATCCTCAACGCCAAGTTCG | TGCCAACGGGTGCTATGAAG |
| E1 | GTGTTTCAGCAGGTGGCGC | CCGGAACAGCCTGAAGAAGTG |
| E2 | AAACGCACTTTCGTGGCAGC | ATCATTGGGGGCACTTGTCG |
| F1 | CAGCATTCTCCTTCTCGGTGTG | CACGGTCCTCTCTTCATCCAAGTAG |
| F2 | AACCAATGGGTGAAGGAGGC | AAGTGGATGGCGTTGAGGAGAAGC |
| G1 | GAGCCTCCTCAAATCCAAATGC | TTGTTGCGACCTTCCCTTCG |
| I2 | AAGTGTATGTTTCCCAAGTGACGC | GGATGTGTATGCCAGTTGATGTTG |
|  |  |  |
