# Supplementary material for: Host cell and viral protease targets of human SERPINs identified by in silico docking
Source: EMBO J. 2025 Sep 8;44(20):5755–84. doi: 10.1038/s44318-025-00546-6 (PMC12528359; doi:10.1038/s44318-025-00546-6)
Supplement: Supplementary file 12 — Expanded View Figures [file 44318_2025_546_MOESM12_ESM.pdf]

Expanded View Figures

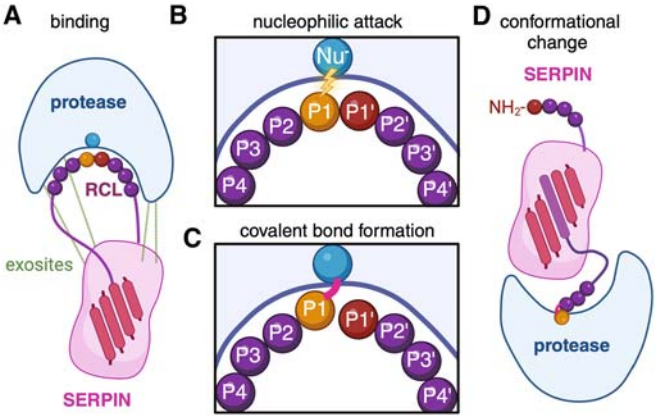

Figure EV1. Molecular mode of action for inhibitory SERPINs.

(**A**) A SERPIN binds to a target protease by inserting its reactive center loop (RCL, purple line) into the protease's catalytic center. This *binding step* is facilitated by the 3D fit of a given RCL into the catalytic center of the protease and can be enhanced by the formation of secondary binding sites ("exosites", green dashed lines). (**B**) The RCL core sequence (named P4-P4') mimics the core sequence of the canonical protease substrate. *Nucleophilic attack* by the protease cleaves the SERPIN at the P1-P1' bond. (**C**) Protease and SERPIN form a *covalent complex* (acyl-enzyme intermediate). (**D**) The ensuing rapid and significant *conformational change*, where the RCL attached to the protease inserts itself into a ß-sheet center, prompts the formation of a stable inhibitory complex between SERPIN and protease, akin to a "mousetrap".

 © The Author(s)

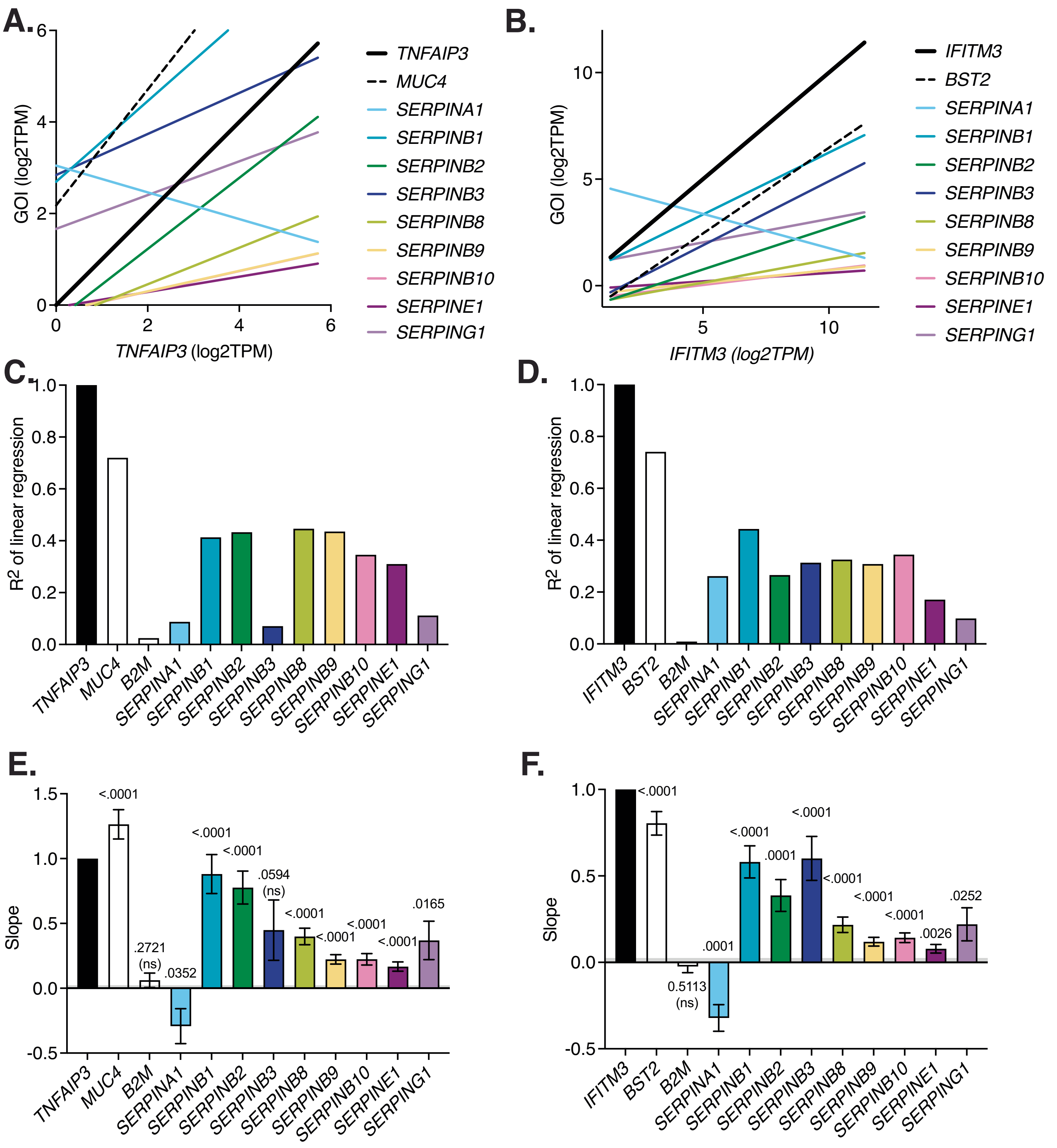
A.
GOI (log2TPM)
TNFAIP3 (log2TPM)
TNFAIP3
MUC4
SERPINA1
SERPINB1
SERPINB2
SERPINB3
SERPINB8
SERPINB9
SERPINB10
SERPINE1
SERPING1
B.
IFITM3 (log2TPM)
IFITM3
BST2
C.
R2 of linear regression
B2M
D.
E.
Slope
<.0001
.2721 (ns)
.0352
.0594 (ns)
.0165
F.
0.5113 (ns)
.0001
.0026
.0252

Figure EV2. Correlation analysis of SERPIN mRNA with TNF-alpha or type I interferon-induced genes.

(**A**) Linear regression plot with correlation analysis of select mRNA levels in reference to canonical TNF-alpha-regulated gene TNFAIP3. MUC4, TNF-alpha-regulated gene positive control. (**B**) Linear regression plot with correlation analysis of select mRNA levels in reference to canonical type I-interferon-regulated gene IFITM3. BST-2, Interferon-regulated gene positive control. (**C**, **D**) R2 values of the linear regression plots from (**A**, **B**), respectively. (**E**) Magnitude of slopes of linear regression from (**A**) and linear regression statistics testing that the slope is significantly non-zero. Mean +/− SD. Exact P values MUC4 $P = 3.85 \times 10^{-15}$; B2M $P = 0.2721$; SERPINB8 $P = 8.4 \times 10^{-8}$; SERPINE1 $P = 2.21 \times 10^{-5}$; SERPINB2 $P = 1.55 \times 10^{-7}$; SERPINB1 $P = 3.63 \times 10^{-7}$; SERPING1 $P = 0.0165$; SERPINB3 $P = 0.0594$; SERPINA1 $P = 0.0352$; SERPINB9 $P = 1.38 \times 10^{-7}$; SERPINB10 $P = 5.65 \times 10^{-6}$; BST2 $P = 1.37 \times 10^{-7}$; MUC16 $P = 1.3 \times 10^{-10}$; IFITM3 $P = 1.19 \times 10^{-9}$, $n = 3$ technical replicates (**F**). Magnitude of slopes of linear regression from (**B**) and linear regression statistics testing that the slope is significantly non-zero. GOI, gene of interest; ns: not significant. Exact P values SERPINB8 $P = 1.26 \times 10^{-5}$; SERPINE1 $P = 0.00258$; SERPINB2 $P = 1.1 \times 10^{-4}$; SERPINB1 $P = 9.85 \times 10^{-8}$; SERPING1 $P = 0.0252$; SERPINB3 $P = 1.98 \times 10^{-5}$; SERPINA1 $P = 1.26 \times 10^{-4}$; SERPINB9 $P = 2.39 \times 10^{-5}$; SERPINB10 $P = 6.05 \times 10^{-6}$; BST2 $P = 5.61 \times 10^{-16}$, $n = 3$ technical replicates. *$P < 0.05$,**$P < 0.005$, ***$P < 0.0001$, ****$P < 0.00001$.

© The Author(s)

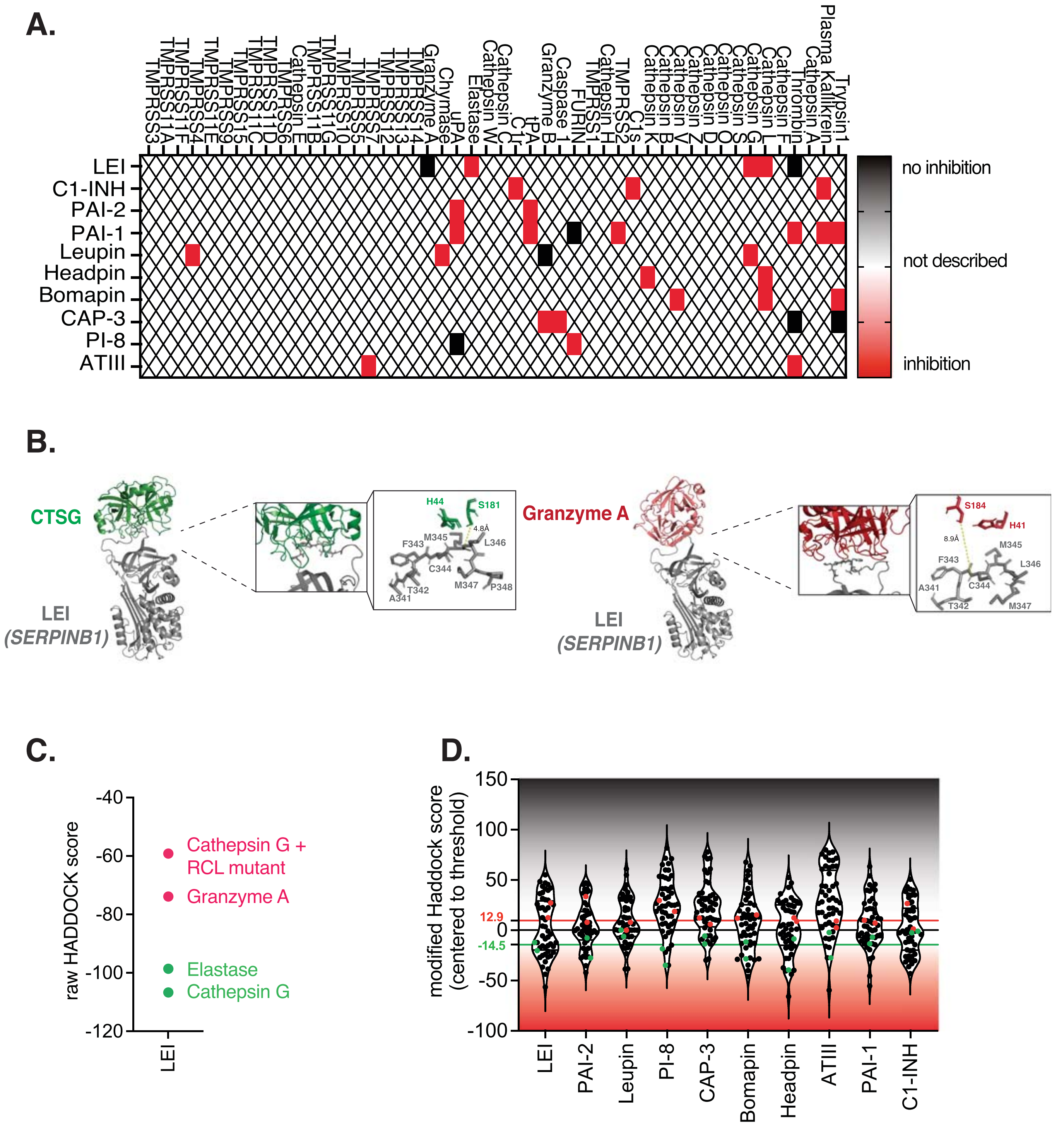
A.
TMPRSS3
TMPRSS11A
TMPRSS11F
TMPRSS4
TMPRSS11E
TMPRSS9
TMPRSS15
TMPRSS11C
TMPRSS11D
TMPRSS6
Cathepsin E
TMPRSS11B
TMPRSS11G
TMPRSS10
TMPRSS5
TMPRSS7
TMPRSS12
TMPRSS13
TMPRSS14
Granzyme A
Chymase
uPA
Elastase
Cathepsin W
Cathepsin C
C1r
tPA
Granzyme B
Caspase 1
FURIN
TMPRSS1
Cathepsin H
TMPRSS2
C1s
Cathepsin K
Cathepsin B
Cathepsin V
Cathepsin Z
Cathepsin D
Cathepsin O
Cathepsin S
Cathepsin G
Cathepsin L
Cathepsin F
Thrombin
Cathepsin A
Plasma Kallikrein
Trypsin1
LEI
C1-INH
PAI-2
PAI-1
Leupin
Headpin
Bomapin
CAP-3
PI-8
ATIII
no inhibition
not described
inhibition
B.
CTSG
H44
S181
4.8Å
F343
M345
L346
C344
T342
M347
P348
A341
LEI
(SERPINB1)
Granzyme A
S184
H41
8.9Å
M345
F343
L346
A341
C344
T342
M347
LEI
(SERPINB1)
C.
raw HADDOCK score
-40
-60
-80
-100
-120
Cathepsin G + RCL mutant
Granzyme A
Elastase
Cathepsin G
LEI
D.
modified Haddock score (centered to threshold)
150
100
50
0
-50
-100
12.9
-14.5
LEI
PAI-2
Leupin
PI-8
CAP-3
Bomapin
Headpin
ATIII
PAI-1
C1-INH

Figure EV3. Establishing the in-silico screen for identifying SERPIN-protease pairs.

(**A**) Previously described SERPIN-protease pairs in the format of Fig. 3B. (**B**) 3D structure overview featuring leukocyte elastase inhibitor (LEI, encoded by *SERPINB1*, gray) docked to Cathepsin G (CTSG, green). Detailed interface view showing LEI RCL and protease active sites. Zoom-ins reveal residues involved in catalysis: nucleophile S181 and its general base H44 are positioned opposite LEI's M345↓L346 with S181 and M345 in close proximity (4.8 A). LEI (gray) docked to Granzyme A (red). Detailed interface view showing LEI RCL and protease active sites. Zoom-ins reveal residues involved in catalysis: nucleophile S184 and its general base H41 are positioned opposite LEI F343↓C344, with S184 and M344 further apart (8.9 A). (**C**) Raw HADDOCK scores for SERPINB1 with positive controls (Cathepsin G, Elastase) or negative controls (RCL mutant + Elastase, Granzyme A). More negative scores indicate favorable binding. (**D**) Violin Plots of normalized HADDOCK scores for $n = 10$ SERPINs docked to $n = 48$ proteases and to respective negative and positive controls (listed in "Methods"). LEI, leukocyte elastase inhibitor encoded by *SERPINB1*; PAI-2, plasminogen activator inhibitor 2 encoded by *SERPINB2*; Leupin, encoded by *SERPINB4*; PI-8, protease inhibitor 8 encoded by *SERPINB8*, CAP-3, cytoplasmic anti-protease 3 encoded by *SERPINB10*, Bomapin encoded by *SERPINB10*, Headpin encoded by *SERPINB13;* ATIII, antithrombin 3 encoded by *SERPINC1;* PAI-1, plasminogen activator inhibitor 1 encoded by *SERPINE1;* C1-INH, C1 inhibitor encoded by *SERPING1*. Black line, mean of all 480 values set to 0; red line, mean negative control; red background, range for predicted non-binders; green line, mean positive control; green background, range for predicted binders; red data points, negative controls; green data points, positive controls. Raw HADDOCK scores and normalization in Dataset EV2.

 © The Author(s)

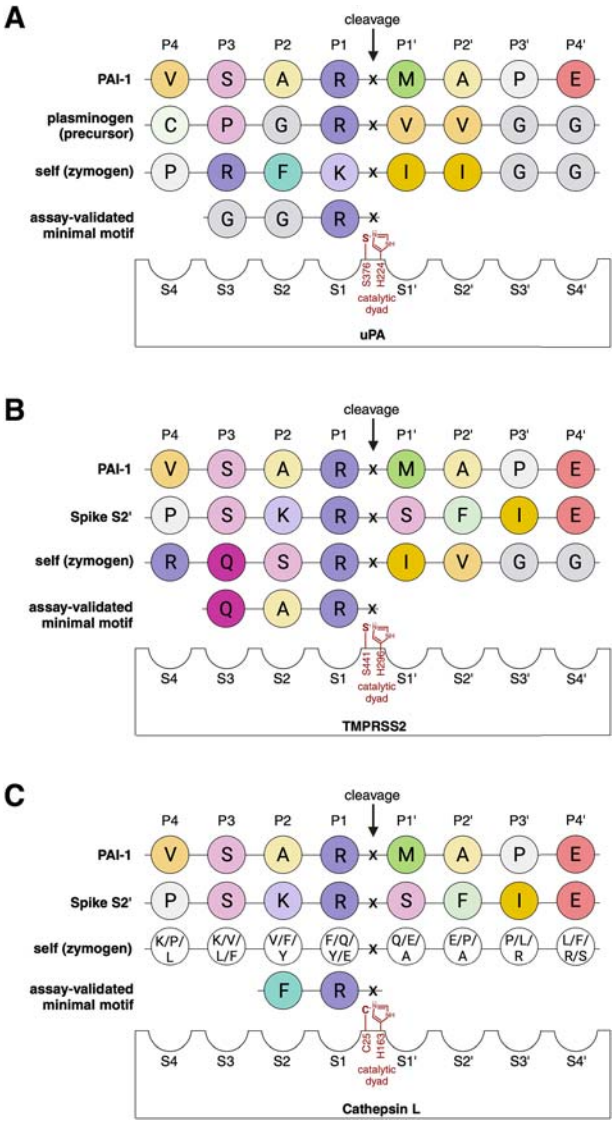

Figure EV4. Schematic representation of substrate P4-P4′ motifs opposite proteases′ catalytic pockets S4-S4′ for uPA, TMPRSS2 and Cathepsin L.

(**A–C**) Schematic of fit of substrate residues into protease pockets for uPA, TMPRSS2, and cathepsin L, respectively. Substrates include zymogen self-cleavage region, canonical or previously described substrate, PAI-1 and fluorophore-linked peptide substrate used in this study. Amino acids are colored according to their side chain chemistry (Unipro UGENE): basic (R, K) litmus blue with R being more basic and darker; acidic (E, D) litmus red with more acidic being darker; hydrophobic (I, L, V, A), yellow with intensity corresponding to hydrophobic character; sulfur-containing (C, M) green; aromatic (F, Y, W) in teal; polar (N, Q, S, T) magenta/pink with darker coloring for more polarity; non-polar glycine (G) in dark gray and proline (P) in light gray. Assembled from references (Koga et al, 1990; Menard et al, 1998; Rossignol et al, 2004; Shrimp et al, 2020) and UNIPROT.

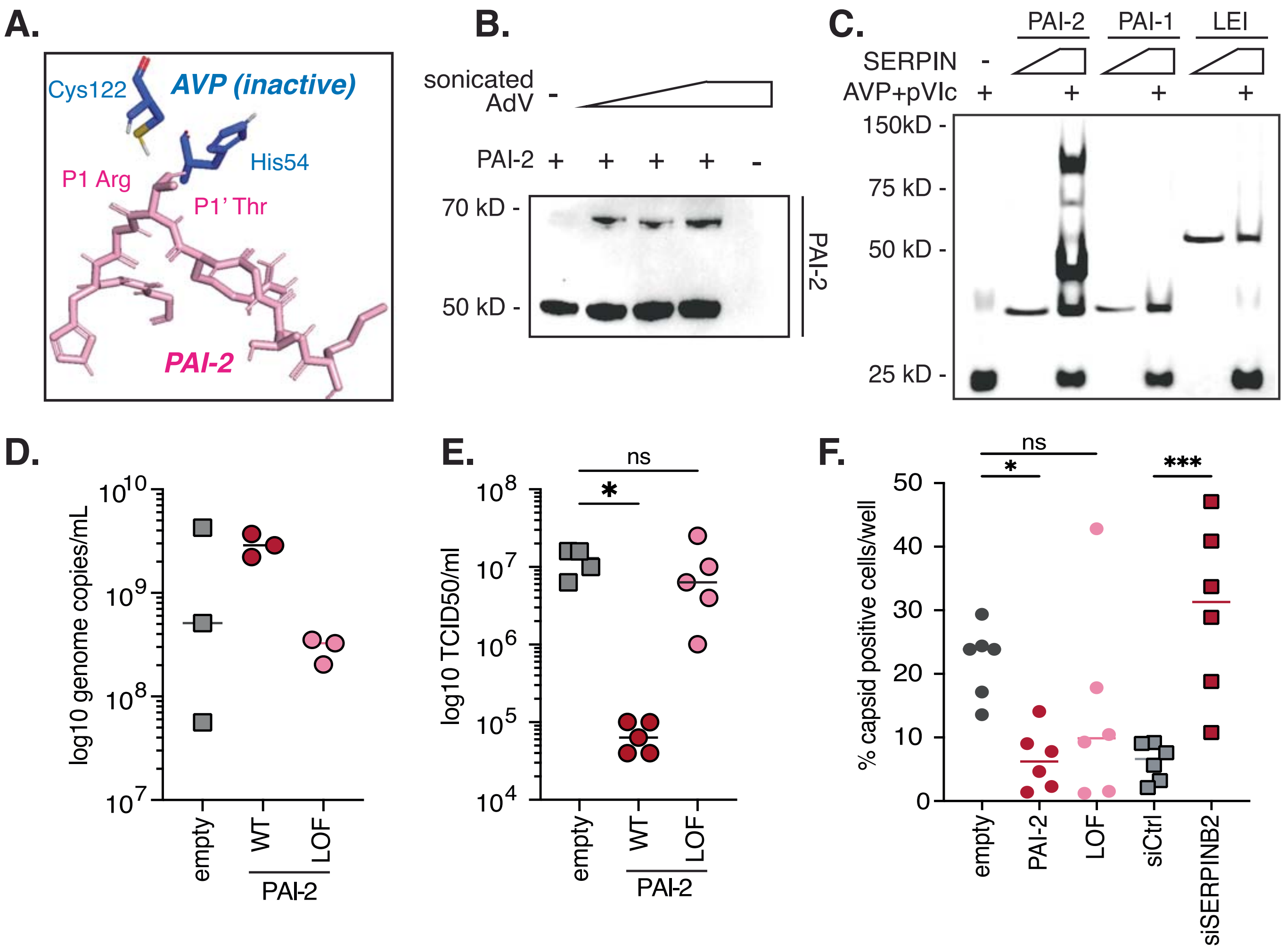

Figure EV5. PAI-2 binding assays to inactive AVP and expression quality control.

(**A**) Docking structure of top-scoring complex for PAI-2 with inactive AVP (structure solved without pVIc binding). (**B**) Western blot of AVP from sonicated adenovirus particles (23 kDa, not visible; bound to pVIc and DNA and thus in its most active form) mixed with rPAI-2 (47 kDa) and probed for PAI-2. (**C**) Silver-stained SDS-PAGE of AVP+pVIC incubated with PAI-2, PAI-1, or LEI in increasing concentrations (1-7.5 ng). (**D**) Genome copy numbers as quantified by qPCR of supernatants derived from HAdV5-infected A549s at 72hpi, transduced with either empty PAI-2 or LoF expression construct, $n = 3$. 6 h. (**E**) TICD50 of supernatants derived from HAdV5-infected A549s, transduced with either empty, PAI-2 or LoF expression constructs. Exact P values: empty vs. PAI-2 $P = 0.0271$, empty vs. LOF $P = 0.7372$, $n = 5$. (**F**) AdV capsid protein-positive HEK-293T cells per well after transfection with either empty, PAI-2 or LOF expression constructs, or control siRNA, SERPINB2 siRNA, followed by 72 h AdV5-GFP infection. Western blot and % infected. Statistical analysis by ANOVA, $^{*}P < 0.05$; ns, non-significant. Exact P values: empty vs. PAI-2 $P = 0.0264$, empty vs. LOF $P = 0.4381$, siCtrl vs siSERPINB2 $P = 0.0004$, $n = 6$.

 © The Author(s)
